# Kawasaki Disease-Associated Pancreatitis in an Adolescent: A Case Report and Literature Review

**DOI:** 10.3390/pediatric18010022

**Published:** 2026-02-04

**Authors:** Akihiro Ichiki, Keisuke Takata, Tadashi Moriwake

**Affiliations:** Department of Pediatrics, NHO Iwakuni Clinical Center, Yamaguchi 740-8510, Japan

**Keywords:** Kawasaki disease, pancreatitis, systemic vasculitis, intravenous immunoglobulin

## Abstract

Background: Pancreatic involvement in Kawasaki disease (KD) is rare. Case presentation: A 13-year-old adolescent presented with severe epigastric pain, elevated pancreatic enzyme levels, and conjunctival injection, but he lacked a fever and the other classic features of KD. The patient was initially diagnosed with acute pancreatitis and treated conservatively. As his abdominal pain improved, mucocutaneous findings emerged, leading to a diagnosis of complete KD. A literature review was conducted to summarize reported cases of KD-associated pancreatitis. This review highlights the older age of affected patients, the variability in the timing of pancreatitis onset, and a tendency toward delayed diagnosis. Conclusions: Pancreatic involvement, including pancreatitis, can occur before typical mucocutaneous features and should be considered in older children and adolescents presenting with unexplained abdominal pain and pancreatic enzyme elevation. Increased awareness of this atypical presentation may help reduce diagnostic delay and support timely management.

## 1. Introduction

Kawasaki disease (KD) is an acute, self-limiting systemic vasculitis that primarily affects medium-sized arteries. Gastrointestinal manifestations, including abdominal pain, vomiting, hepatic dysfunction, and gallbladder hydrops, are relatively common [1]. However, pancreatic involvement is exceedingly rare. Importantly, when pancreatitis occurs as an initial manifestation before the development of classic mucocutaneous features, the diagnosis of KD can be particularly challenging and may be delayed. To date, only a limited number of cases of KD-associated pancreatitis have been reported, and the diagnostic approach and optimal management are not well established. Here, we report the case of an adolescent with KD, whose initial presentation was acute pancreatitis without classic KD manifestations. This case highlights the diagnostic challenge posed when pancreatitis is the initial manifestation of KD and underscores the importance of considering KD in children and adolescents presenting with unexplained pancreatic involvement.

## 2. Case Presentation

A 13-year-old Japanese boy was referred to our hospital with epigastric pain lasting for 1 day. Nine days before admission, he had developed fever and headache. Conjunctival injection appeared thereafter, and new-onset epigastric pain appeared one day before admission. He had no significant past or family history and no known allergies. He had normal growth and development and had received all routine childhood vaccinations.

On admission, he had a heart rate of 80 beats/min, blood pressure of 130/80 mmHg, temperature of 37.2 °C, and oxygen saturation of 99% on ambient air. Physical examination revealed marked epigastric tenderness without muscular defense or rebound tenderness. The patient was afebrile but showed conjunctival injection, without other mucocutaneous features of KD. Laboratory tests showed leukocytosis (15.1 × 10^3^/μL) with neutrophilia (89.2%) and elevated C-reactive protein levels (3.8 mg/dL). Serum amylase (334 U/L), pancreatic amylase (61 U/L), lipase (77 U/L), aspartate aminotransferase (46 U/L), and alanine transaminase (56 U/L) levels were elevated. Fasting, continuous intravenous fluid replacement, antibiotics, and analgesics were initiated for suspected acute pancreatitis. Intermittent epigastric pain persisted, and fever developed on the third day of hospitalization; however, no other gastrointestinal symptoms were observed. There was no evidence of gallstones, biliary dilatation, or any pancreatic abnormalities on imaging, including ultrasound and magnetic resonance imaging (MRI). Rapid antigen tests for COVID-19, influenza virus, human metapneumovirus, respiratory syncytial virus, group A *Streptococcus*, and adenovirus were negative. Polymerase chain reaction (PCR) tests for measles and rubella showed negative results. Serological testing indicated no previous infection with Epstein–Barr virus (EBV) or past infection with cytomegalovirus (CMV). An interferon-gamma release assay (IGRA) was negative. Serum IgG4 levels were within the normal range.

After ulinastatin (UTI) was initiated, the abdominal pain resolved, and the serum total amylase, pancreatic amylase, and lipase levels decreased from their peak values of 334 IU/L, 95 IU/L, and 126 IU/L, respectively. However, fever and conjunctival injection persisted, and a rash subsequently appeared on the trunk and limbs. Echocardiography demonstrated dilation of the proximal right coronary artery (4.3 mm, Z-score 2.7) and the left anterior descending artery (4.0 mm, Z-score 2.9), fulfilling the criteria for coronary artery aneurysm. Upon re-interviewing the patient and his family, cervical lymphadenopathy was reported to have occurred prior to admission, although it was not present at the time of hospitalization. He was diagnosed with complete KD on hospital day 9 (illness day 18 from the onset of fever) after persistent fever since hospital day 3, the appearance of clinical features, and the detection of coronary artery aneurysms. Despite the initial intravenous immunoglobulin (IVIG), aspirin, UTI, and cyclosporin A (CsA), he remained febrile and required infliximab (IFX), additional IVIG, and methylprednisolone pulse therapy. He subsequently received tapering corticosteroid therapy as maintenance treatment. No re-elevation of pancreatic enzymes or relapse of pancreatitis was observed during follow-up, and the coronary artery aneurysms remained stable.

## 3. Discussion

KD rarely presents with pancreatic involvement, with only a limited number of previously reported cases [2,3,4,5,6,7,8,9,10,11,12,13,14,15]. Our adolescent patient presented with severe epigastric pain, marked elevation of pancreatic enzymes, and conjunctival injection at admission, but he lacked fever and other classic KD features. These atypical findings initially led to a diagnosis of acute pancreatitis and delayed the recognition of KD, illustrating a key diagnostic challenge.

A narrative literature review was conducted to identify previously reported cases of KD associated with pancreatitis. We searched the PubMed database for English-language articles published up to September 2025 using the search terms “Kawasaki disease” and “pancreatitis.” In addition, the reference lists of relevant articles were manually reviewed to identify additional reports not indexed in PubMed.

Previous reports have indicated that the timing of pancreatic involvement in KD varies. Although abdominal pain is the most frequent symptom of acute pancreatitis, some patients are asymptomatic and identified only through elevated levels of pancreatic enzymes. Our review found that 9 of the 17 reported cases (53%) experienced abdominal pain before KD was clinically diagnosed, and IVIG was initiated between illness days 7 and 22, with a median of day 9 [3,8,9,11,12,13]. Because timely IVIG administration is crucial for reducing the risk of coronary artery aneurysms, such diagnostic delays may have important clinical implications [16]. Notably, pancreatitis-associated KD tended to occur in older children and adolescents, with 12 out of 17 patients (71%) being ≥5 years old, older than the typical peak age of KD. When abdominal pain precedes mucocutaneous manifestations, clinicians may prioritize abdominal emergencies or primary pancreatitis, contributing to diagnostic delays, particularly in older children and adolescents in whom KD is less commonly suspected. Among patients whose abdominal pain developed before KD was clinically diagnosed, the number of KD symptoms present at the time of pain onset ranged widely (1–5 symptoms), with no specific mucocutaneous sign appearing earlier than the others. This heterogeneity in the early clinical presentation of KD-associated pancreatitis likely further contributes to diagnostic difficulty. The clinical characteristics, laboratory findings, and treatment responses of the previously reported 16 cases, together with those of our patient, are summarized in Table 1.

Acute pancreatitis in children can be caused by a wide variety of factors, including congenital or anatomical abnormalities, infections, drugs, trauma, systemic inflammatory diseases, metabolic disorders, and nutritional factors [17]. In the present case, the patient presented with characteristic epigastric pain suggestive of acute pancreatitis. However, pancreatic enzyme elevation was mild, and imaging findings were unremarkable; therefore, the International Study Group of Pediatric Pancreatitis: In Search for a Cure (INSPPIRE) diagnostic criteria for acute pancreatitis were not strictly met [18]. In contrast, at the time of diagnosis, the 2015 Japanese clinical guidelines for acute pancreatitis did not specify a strict threshold for pancreatic enzyme elevation; therefore, acute pancreatitis was clinically suspected in this case [19].

Abdominal MRI revealed no evidence of gallstones, biliary dilatation, or triglycerides. There was no history of medication or trauma, and serological tests for common viral infections (including EBV, CMV, and mycoplasma) were negative. Although other inflammatory or systemic diseases can cause pancreatitis, no clinical features or laboratory findings indicated the presence of these conditions.

Conjunctival injection was observed at admission; however, the absence of a persistent fever and other mucocutaneous manifestations made the diagnosis of KD challenging at presentation. The patient gradually developed the characteristic features of KD and was diagnosed with complete KD according to the Japanese diagnostic guidelines [20]. Although he presented with mucocutaneous findings, fever, and coronary involvement, there were no signs of circulatory dysfunction or coagulopathy, and SARS-CoV-2 antigen testing was negative with no known exposure to COVID-19. These distinctions did not fulfill the World Health Organization (WHO) criteria for multisystem inflammatory syndrome (MIS-C). The presence of conjunctival injection at the time of abdominal pain further supported the idea that pancreatic involvement may have been an early manifestation of KD. Taken together, these findings suggested that the pancreatic involvement was most consistent with KD-associated pancreatitis.

However, the pathophysiology of pancreatic involvement in KD has not been fully elucidated. Several pathological studies suggest that pancreatic involvement may reflect the same immune-mediated vasculitic process underlying KD rather than representing a purely incidental finding. Amano et al. reported that autopsies of patients with KD who died before the introduction of IVIG therapy revealed pancreatitis in 22% of cases and inflammation of the pancreatic duct in 53% of cases [21]. Rowley et al. reported a distinctive pattern of IgA plasma cell infiltration in both vascular and non-vascular tissues in acute fatal KD, including the coronary arteries, pancreatic ducts, and kidneys [22]. Moreover, CD8 + T lymphocytes and macrophages have been identified in the pancreatic islets, displaying an inflammatory pattern similar to that observed in coronary artery aneurysms [23]. These findings support the hypothesis that pancreatic inflammation in KD is primarily immune-mediated and may share a common vasculitic mechanism with coronary artery lesions. Nevertheless, important uncertainties remain, including whether pancreatic involvement represents a primary target of vasculitis or a secondary manifestation of systemic inflammation, as well as its true clinical significance. Because pancreatic involvement may be asymptomatic or overshadowed by other systemic features, it may also be underrecognized in routine clinical practice.

The therapeutic strategies for KD-associated pancreatitis are not well established. In previously reported cases, pancreatic involvement was generally managed conservatively with supportive care, including fasting and intravenous fluid therapy. Regarding KD, initial treatment commonly consisted of IVIG. Among the 15 reported cases, including ours, 13 were treated with IVIG; however, IVIG resistance occurred in 4 patients [6,8,24,25]. Because these observations are based on a small number of published cases and are subject to publication bias, they should be interpreted with caution. In the present case, UTI, a protease inhibitor used for acute pancreatitis and listed as an off-label option in the Japanese KD guidelines, improved the pancreatic symptoms [20]. However, UTI did not prevent the subsequent emergence of classic KD manifestations. This suggests that UTI improved pancreatic injury but did not control the underlying systemic vasculitis. In our case, corticosteroid therapy was introduced after refractoriness to IVIG, IFX, and CsA, with subsequent clinical improvement. Taken together, these findings suggest that pancreatitis as an atypical presentation of KD may be associated with IVIG resistance, and this raises the hypothesis that methylprednisolone pulse therapy may be considered in selected patients with KD and pancreatic involvement.

## 4. Conclusions

Pancreatic involvement, including pancreatitis, may precede typical features of KD and contribute to diagnostic delay, especially in older children and adolescents. Awareness of this atypical presentation may facilitate earlier recognition of KD in patients with unexplained pancreatitis.

## Figures and Tables

**Table 1:** Summary of published data on Kawasaki disease with pancreatic involvement.

Reference	Age (Years)	Sex	Pancreatitis Category (1–3)	KD Symptom Count at Abdominal Pain Onset (0–6)	Serum Total Amylase (IU/L)	Serum Lipase (IU/L)	Imaging Showed Pancreas Involvement	Timing of Administration IVIG After Onset of Fever (Days)	IVIG Response * (Yes/No/ND)	Cardiac Involvement
Stoler et al. [2]	5	M	1	NA	197	U	Yes	ND	ND	Yes
Stoler et al. [2]	16	M	3	NA	356	U	Yes	ND	ND	No
Lanting et al. [3]	5	M	2	1	1143	633	Yes	7	Yes	No
Reynaud et al. [4]	18	M	1	NA	241	1678	Yes	12	Yes	No
Asano et al. [5]	1	M	1	NA	407	89	No	6	Yes	No
Cherry et al. [6]	3	M	3	NA	465	3224	Yes	4	No	Yes
Cherry et al. [6]	6	M	3	NA	193	420	Yes	5	No	Yes
Prokic et al. [7]	6	M	2	1	168	95	Yes	7	Yes	No
Jimenez et al. [8]	10	F	2	1	49	1000	No	9	No	No
Albert et al. [9]	19	M	2	5	U	1031	Yes	22	Yes	No
Mitani et al. [10]	3	F	3	NA	210	51	Yes	6	No	No
Botti et al. [11]	3	M	2	3	1369	1330	No	9	Yes	No
Wu et al. [12]	8	F	2	2	61	3322	Yes	8	Yes	No
Tyagi et al. [13]	11	M	2	3	324	249	Yes	U	Yes	No
Das et al. [14]	2	F	1	NA	U	495	Yes	11	Yes	No
Barakat et al. [15]	21	M	2	5	U	U	Yes	U	Yes	Yes
Present case	13	M	2	2	334	126	No	18	No	Yes

1 = asymptomatic elevation of pancreatic enzymes. 2 = pancreatitis occurring before the diagnosis of KD. 3 = pancreatitis occurring after the diagnosis of KD. * IVIG response was defined as follows: “Yes” was assigned to cases in which fever resolved after the first IVIG infusion without any additional therapy. “No” was assigned to cases that required additional treatment after the initial IVIG dose. “ND (not done)” was used when IVIG was not administered. Insufficient information was available to determine the IVIG response. IVIG, intravenous immunoglobulin; NA, not applicable; ND, not done; U, unknown.

## Data Availability

The data presented in this study are available on request from the corresponding author due to privacy restrictions.

## Abbreviations

The following abbreviations are used in this manuscript:

CMV	Cytomegalovirus
CsA	Cyclosporin A
EBV	Epstein–Barr virus
IFX	Infliximab
IGRA	Interferon-gamma release assay
INSPPIRE	International Study Group of Pediatric Pancreatitis: In Search for a Cure
IVIG	Intravenous immunoglobulin
KD	Kawasaki disease
MIS-C	Multisystem inflammatory syndrome
MRI	Magnetic resonance imaging
PCR	Polymerase chain reaction
UTI	Ulinastatin
WHO	World Health Organization
